# Predicting intraoperative 5-ALA-induced tumor fluorescence via MRI and deep learning in gliomas with radiographic lower-grade characteristics

**DOI:** 10.1007/s11060-024-04875-0

**Published:** 2024-11-19

**Authors:** Eric Suero Molina, Ghasem Azemi, Zeynep Özdemir, Carlo Russo, Hermann Krähling, Alexandra Valls Chavarria, Sidong Liu, Walter Stummer, Antonio Di Ieva

**Affiliations:** 1 Department of Neurosurgery, University Hospital Münster, Albert-Schweitzer-Campus 1, A1, 48149 Münster, Germany; 2 Computational NeuroSurgery (CNS) Lab, Macquarie Medical School, Macquarie University, 75 Talavera Road, Sydney, NSW 2109 Australia; 3 Macquarie Neurosurgery & Spine, Macquarie University Hospital, Sydney, Australia; 4 Clinic for Radiology, University Hospital Münster, Münster, Germany

**Keywords:** 5-ALA, Fluorescence-guided resection, Lower-grade gliomas, Deep learning, Autoencoder

## Abstract

**Purpose:**

Lower-grade gliomas typically exhibit 5-aminolevulinic acid (5-ALA)-induced fluorescence in only 20–30% of cases, a rate that can be increased by doubling the administered dose of 5-ALA. Fluorescence can depict anaplastic foci, which can be precisely sampled to avoid undergrading. We aimed to analyze whether a deep learning model could predict intraoperative fluorescence based on preoperative magnetic resonance imaging (MRI).

**Methods:**

We evaluated a cohort of 163 glioma patients categorized intraoperatively as fluorescent (n = 83) or non-fluorescent (n = 80). The preoperative MR images of gliomas lacking high-grade characteristics (e.g., necrosis or irregular ring contrast-enhancement) consisted of T1, T1-post gadolinium, and FLAIR sequences. The preprocessed MRIs were fed into an encoder-decoder convolutional neural network (U-Net), pre-trained for tumor segmentation using those three MRI sequences. We used the outputs of the bottleneck layer of the U-Net in the Variational Autoencoder (VAE) as features for classification. We identified and utilized the most effective features in a Random Forest classifier using the principal component analysis (PCA) and the partial least square discriminant analysis (PLS-DA) algorithms. We evaluated the performance of the classifier using a tenfold cross-validation procedure.

**Results:**

Our proposed approach's performance was assessed using mean balanced accuracy, mean sensitivity, and mean specificity. The optimal results were obtained by employing top-performing features selected by PCA, resulting in a mean balanced accuracy of 80% and mean sensitivity and specificity of 84% and 76%, respectively.

**Conclusions:**

Our findings highlight the potential of a U-Net model, coupled with a Random Forest classifier, for pre-operative prediction of intraoperative fluorescence. We achieved high accuracy using the features extracted by the U-Net model pre-trained for brain tumor segmentation. While the model can still be improved, it has the potential for evaluating when to administer 5-ALA to gliomas lacking typical high-grade radiographic features.

**Graphical abstract:**

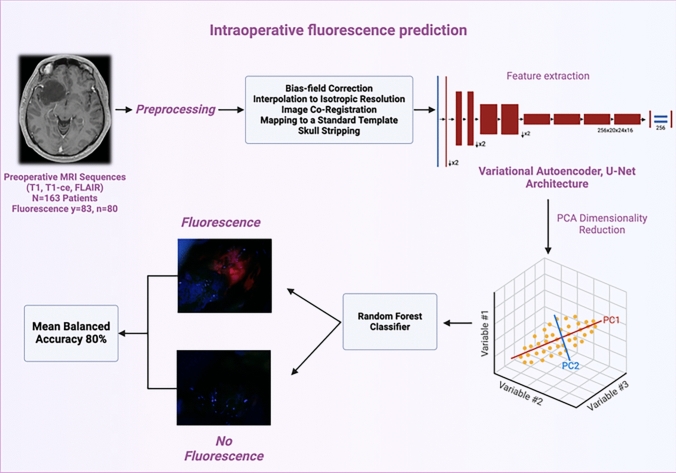

**Supplementary Information:**

The online version contains supplementary material available at 10.1007/s11060-024-04875-0.

## Introduction

Gliomas are the most prevalent malignant primary brain tumors in adults [1, 2]. They originate from the glial cells in the central nervous system [3]. Lower-grade gliomas (LGG) are characterized by molecular features, such as accumulation of 2-hydroxyglutarate caused by the isocitrate dehydrogenase (IDH) gene mutation [4], which plays a significant role in their classification and prognosis. LGG, classified as WHO Grade 2 or 3 gliomas, grow slower and are less aggressive than their high-grade counterparts [5]. Nevertheless, due to their infiltrative nature and potential for malignant transformation, their treatment is challenging [6].

Fluorescence-guided surgery (FGS) using 5-aminolevulinic acid (5-ALA) has become a cornerstone technique for maximizing the removal of high-grade gliomas (HGG) [7, 8]. 5-ALA is a natural precursor in heme biosynthesis [9] that, when orally administered, leads to the accumulation of fluorescent Protoporphyrin IX (PpIX) in HGG tissue [10–13].

Intraoperative visualization of LGG tissue poses another challenge during tumor resection. While 5-ALA-guided FGS is approved for high-grade gliomas, the potential applicability in LGG still warrants exploration. LGG's subtle visual differences from non-tumor tissue can pose challenges for surgical resection and pathologic sampling [14]. They can be challenging to depict on ultrasound and may remain inconspicuous to the naked eye. 20–30% of lower-grade gliomas exhibit fluorescence after 5-ALA administration. This rate can be improved by measuring lower PpIX concentrations using spectroscopy [15–17] or doubling the administered dose of 5-ALA [18]. Up to 55% of all LGGs have malignant foci that are usually assigned to a higher tumor grade and, therefore, require a different adjuvant treatment regimen [19, 20]. These foci generally tend to fluoresce [6, 21]. Accurately identifying these malignant regions during surgery is crucial for delivering malignant tissue for histopathological evaluation and avoiding undergrading. Therefore, FGS has been employed in LGG more to identify these foci rather than to maximize the extent of resection, as these tumors rarely fluoresce homogenously [18].

We aimed to analyze whether a deep learning model could predict intraoperative fluorescence based on preoperative magnetic resonance imaging (MRI).

## Methods

We retrospectively selected patients with lower-grade gliomas who underwent surgery with 5-ALA-mediated FGS and documented the intraoperative fluorescence visibility binarized as fluorescent or non-fluorescent, irrespective of the pattern or intensity of fluorescence. We searched for gliomas lacking high-grade radiographic characteristics, i.e., irregular ring contrast enhancement. MRI protocols included the following sequences: T1, T1-contrast enhanced (T1ce), and FLAIR. As the initial MRI was often performed externally, our database comprised images acquired from 25 MRI scanners (Suppl. Table 1). 5-ALA was administered in a standard dosage of 20 mg/kg four hours before anesthesia induction.

Based on the abovementioned criteria, we selected patients who underwent surgery at the Department of Neurosurgery, University Hospital Münster, Germany, between January 2010 and May 2023. Tumor biopsies included in this study were assessed according to the WHO classifications in effect at the time of surgery—namely, the 2007, 2016, and 2021 guidelines. Importantly, regardless of the final diagnosis, all tumors were assessed as lacking high-grade glioma (HGG) radiographic characteristics on MRI.

The initial search yielded 550 patients, of which 163 fulfilled the inclusion criteria. The cohort was categorized as fluorescent (n = 83) or non-fluorescent (n = 80) tumors (Table 1).

**Table 1:** Demographic information of the patients enrolled in this study

	N = 163	[%]
Sex		
Male	97	60
Female	66	40
Age		
< 40	63	39
40–69	93	57
≥ 70	7	4
Initial diagnosis	91	56
Recurrence	72	44
WHO grade		
1	0	0
2	125	77
3	22	13
4	14	9
N/A	2	1
Tumor type		
Astrocytoma	102	63
Initial diagnosis	67	66
Recurrence	35	34
Oligodendroglioma	46	28
Initial diagnosis	18	39
Recurrence	28	61
Glioblastoma	12	7
Initial diagnosis	5	42
Recurrence	7	58
Varia	3	2

The study was conducted in accordance with the ethical standards outlined in the Declaration of Helsinki and with approval from the local Ethics Committee of the University of Münster (Approval Number: 2023-545-f-S). Due to the retrospective nature of this study, patient consent was not sought.

Our cohort consisted of 60% male (n = 97) and 40% female (n = 66) patients, with most (57%) aged between 40 and 69 years (Table 1). Brain tumors were initially diagnosed in 56% of cases, with 77% classified as WHO Grade 2. Astrocytoma was the most common tumor type, making up 63% of initial diagnoses. Further details on age and tumor diagnosis can be found in Table 1.

### Preprocessing

The preprocessing pipeline for analyzing brain tumors involved steps designed to address challenges posed by MRI variability in images acquired from different scanners [22]. The images were standardized through a preprocessing pipeline involving isotropic transformation, bias field correction, co-registration to T1-T1-ce space, mapping to a brain atlas, and skull stripping.

The preprocessing pipeline was designed to enhance the quality and consistency of the MRI data for subsequent analysis. Firstly, the bias-field correction was applied to mitigate intensity variations caused by non-uniformity in the magnetic field during image acquisition. This step ensures that the intensity values across the images are normalized. Next, interpolation to a uniform resolution, i.e., 1 mm^3^, was performed to standardize the spatial resolution across all MRI sequences and mitigate potential discrepancies in voxel size. Subsequently, co-registration of the T1 and FLAIR sequences to the T1-ce sequence was conducted to ensure spatial alignment between different MRI modalities. Following co-registration, the images were mapped to an anatomical template. This step involved aligning the MRI data to the MNI-152 (Montreal Neurological Institute-152) [23] template to compare brain structures across different subjects. Finally, skull stripping was performed to remove non-brain tissues from the images and reduce potential interference from extraneous tissues. We performed skull stripping with the Advanced Normalization Tools (ANTs) *antsBRainExtration* algorithm [24].

This study used the ANTs software package for various preprocessing tasks, including bias field correction, image resampling, co-registration of T1 and FLAIR images to T1-ce, and skull stripping [25]. The MRI sequences were mapped to the MNI-152 template using the FLIRT (FMRIB’s Linear Image Registration Tool) algorithm available in the FMRIB Software Library (FSL), developed at the Oxford Centre for Functional Magnetic Resonance Imaging of the Brain (FMRIB) [26].

### Feature extraction and selection

This study used a pre-trained Variational Autoencoder (VAE) encoder-decoder convolutional neural network (U-Net) architecture specifically tailored for brain tumor segmentation to extract features using MRI sequences (T1, T1-ce, and FLAIR), as described by Russo et al. [27] and Myronenko [28].

**Fig. 1:**
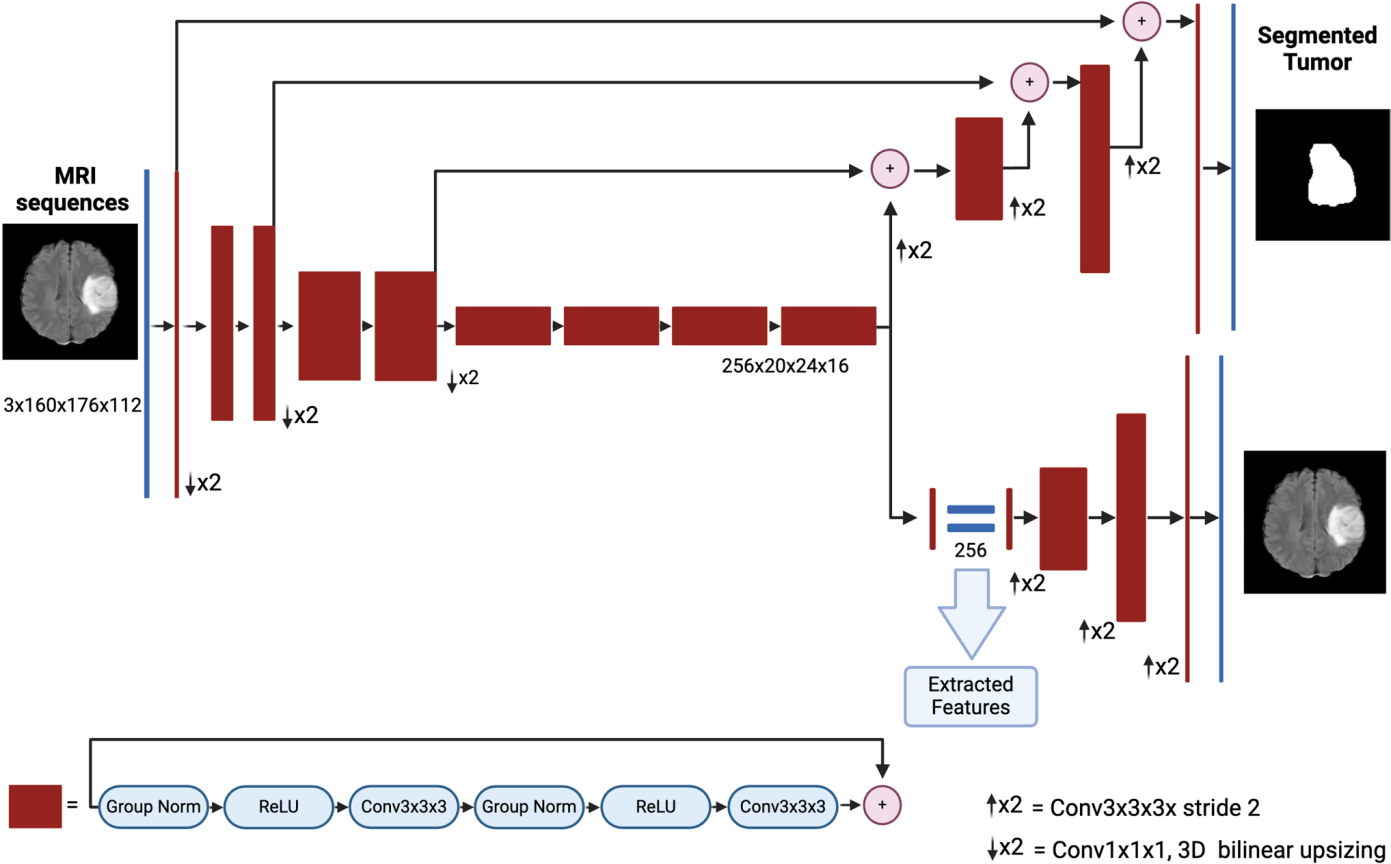
Overview of the pretrained VAE U-Net architecture for MRI-based brain tumor segmentation. This model is based on the one proposed by Russo et al. [27] and employs a Variational Autoencoder U-Net, fine-tuned for segmenting brain tumors from MRI sequences (T1, T1-ce, FLAIR), to accurately capture and reconstruct tumor features. A compact 256-feature vector extracted per subject highlights critical tumor information

A schematic visualization of the model used in this study is shown in Fig. 1. In this model, the primary task of the encoder is to identify crucial lesion characteristics within the input images. The decoder reconstructs these identified features, projecting them into the original image space, resulting in segmentation maps that accurately delineate tumor regions. The features learned by the encoder within the latent space are particularly important to our study, as they encapsulate rich information about the lesions. These features represent the output of the bottleneck layer of the U-Net, signifying the latent space of the VAE for each processed case. Consequently, a feature vector of size 256 was obtained for each subject, as illustrated in Fig. 1. This vector encapsulates the essential characteristics extracted from the MRI sequences and provides a compact yet comprehensive representation of tumor-related information.

Considering the feature vector's high dimensionality relative to the number of samples, principal component analysis (PCA) and partial least squares discriminant analysis (PLS-DA) techniques were employed to reduce the feature space's dimensionality and avoid overfitting.

### Classification and performance evaluation

The selected features underwent classification using a Random Forest (RF) classifier. RF, a versatile ensemble learning method, was chosen for its robustness in handling high-dimensional data and its ability to provide reliable predictions by aggregating the outputs of multiple decision trees. To evaluate performance, this approach was augmented by a tenfold cross-validation procedure.

For every iteration of the cross-validation process, the optimal number of components for PCA and PLS-DA was determined to maximize the overall accuracy. To streamline the search process and avoid excessive computational burden, the maximum number of components for both techniques was constrained to 70. Performance evaluation metrics were recorded across all folds, with mean balanced accuracy, mean sensitivity, and mean specificity serving as the primary measures of classification efficacy.

## Results

**Fig. 2:**
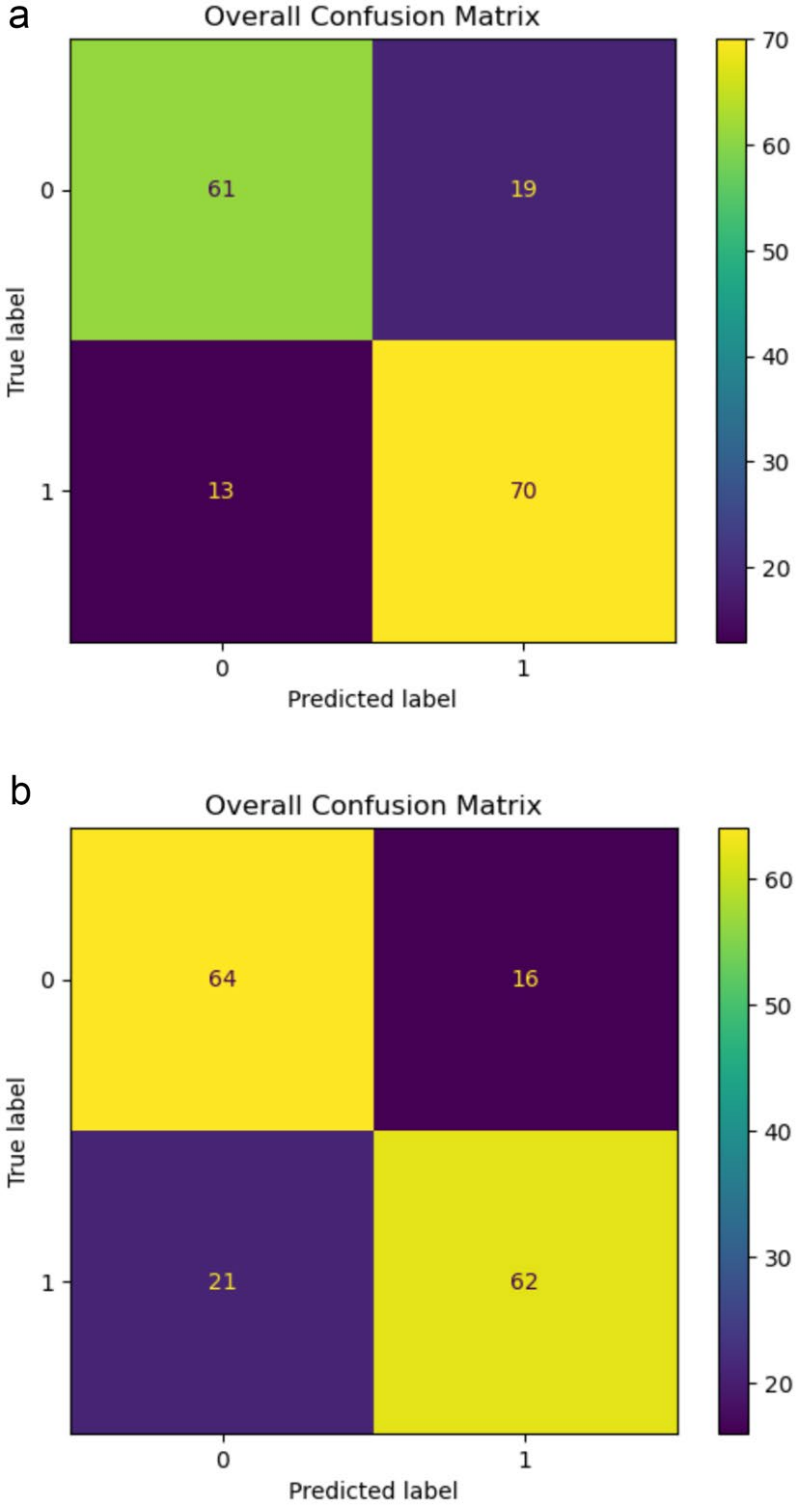
Confusion Matrix for Binary Classification of the random forest classifier for **A** PCA and **B** PLS-DA selected features. It is a tabular representation of the classifier’s performance and shows a summary of the predictions made by a model compared to the actual data labels. The cells represent true negatives (upper left), false positives (upper right), false negatives (lower right), and true positives (lower left)

The overall confusion matrix was computed for each experiment as the cumulative sum of the 10 confusion matrices obtained from each fold. This matrix summarizes the classifier's performance, as illustrated in Fig. 2.

Using the data derived from the confusion matrix, the following performance metrics [29] can be calculated:

$${\text{Mean}}\;{\text{Sensitivity}} = { }\frac{{{\text{TP}}}}{{{\text{TP}} + {\text{FN}}}} = {\text{Acc}}^{ + }$$

$${\text{Mean}}\;{\text{Specificity}} = \frac{{{\text{TN}}}}{{{\text{TN}} + {\text{FP}}}} = {\text{Acc}}^{ - }$$

$${\text{Mean}}\;{\text{Balanced}}\;{\text{Acc}} = \frac{{{\text{Acc}}^{ + } + {\text{Acc}}^{ - } }}{2}{ }$$

$${\text{Precision}} = { }\frac{{{\text{TP}}}}{{{\text{TP}} + {\text{FP}}}}$$

$${\text{Recall}} = \frac{{{\text{TP}}}}{{({\text{TP}} + {\text{FN}})}} = {\text{Acc}}^{ + }$$

$${\text{F}}1 - {\text{Score}} = { }2 \cdot \frac{{{\text{Precision}} \cdot {\text{Recall }}}}{{{\text{Precision}} + {\text{Recall}}}}$$

The classification results obtained from applying two feature selection techniques using an RF classifier are presented. For the features selected through PCA (Fig. 2a), the confusion matrix revealed 131 correctly classified instances, with 61 true negatives and 70 true positives. Conversely, 32 cases were incorrectly classified, comprising 13 false negatives and 19 false positives.

For features refined by PLS-DA (Fig. 2b), the confusion matrix recorded 126 correctly classified instances, including 64 true negatives and 62 true positives. The number of misclassifications summed up to 37, with 21 false negatives and 16 false positives.

Performance measures based on these results indicated that, for PCA-selected features, the mean sensitivity was computed to be 84%, and the mean specificity was calculated at 76%, resulting in a mean balanced accuracy of 80%. For the PLS-DA-selected features, the mean sensitivity was reported as 75%, the mean specificity was 76%, and the resulting mean balanced accuracy was calculated to be 77% (Table 2).

**Table 2:** Comparative performance metrics for PCA versus PLS-DA

Performance metrics		
	PCA	PLS-DA
Mean balanced accuracy	80%	77%
Mean sensitivity	84%	75%
Mean specificity	76%	76%
Precision	0.787	0.795
F1-Score	0.814	0.770

This table compares two multivariate statistical techniques in terms of Mean Balanced Accuracy, Mean Sensitivity, Mean Specificity, Precision, and F1-Score. PCA exhibits higher sensitivity and F1-Score, while PLS-DA has marginally higher precision. Both techniques show equal mean specificity

## Discussion

This work demonstrates the feasibility of using preoperative MRIs to predict intraoperative fluorescence visibility in gliomas without typical high-grade radiographic characteristics using a large heterogeneous database from multiple MRI scanners.

### Preprocessing and data diversity

The preprocessing of MRI data for brain tumor analysis represents a foundational step in building robust models capable of understanding and interpreting complex imaging data. Our approach outlines a preprocessing pipeline to mitigate the variability in MRI scans acquired from different scanners. This diversity, while a challenge, is also an opportunity to enhance the model's ability to generalize across various imaging technologies and external data sources.

MRI data diversity is caused by scanner differences, variations in scanning protocols, and patient-specific factors. These differences can significantly affect the images' intensity values, contrast ratios, and spatial resolutions. Our preprocessing pipeline aims to standardize these images to a standard format, reducing variability and making the data more homogeneous. The ability of a model to generalize well across different imaging technologies is paramount, especially in a clinical setting where MRI scans may come from a myriad of sources.

The diversity of the MRI data in this study presents challenges for model training. Data variability can introduce noise even after standardization, affecting the model's learning process. Generalizing the model across this variability requires careful consideration of the training data. Training the model on a diverse dataset enables it to learn source-independent features, improving its performance.

### Model selection, advanced feature extraction, and validation

Our study employed a VAE integrated with a U-Net model optimized for brain tumor segmentation for advanced feature extraction. By encoding complex data into a lower-dimensional representation, the VAE captures salient features from the U-Net’s deepest layer, refining the information for subsequent classification with a Random Forest model. This innovative strategy leverages deep learning for effective feature extraction while utilizing machine learning for robust decision-making, ultimately enhancing the model's predictive capabilities.

While U-Net’s softmax outputs provide localized pixel-wise probabilities, they lack the depth required for our classification task. Also, although exploring architectures like ResNet, DenseNet, or frameworks such as MONAI and AUCMEDI could yield potential advantages, their implementation would demand significant resources and time. Thus, our approach, utilizing the pretrained U-Net's bottleneck features, effectively balances performance and relevance in predicting intraoperative fluorescence visibility based on preoperative MRIs.

**Fig. 3:**
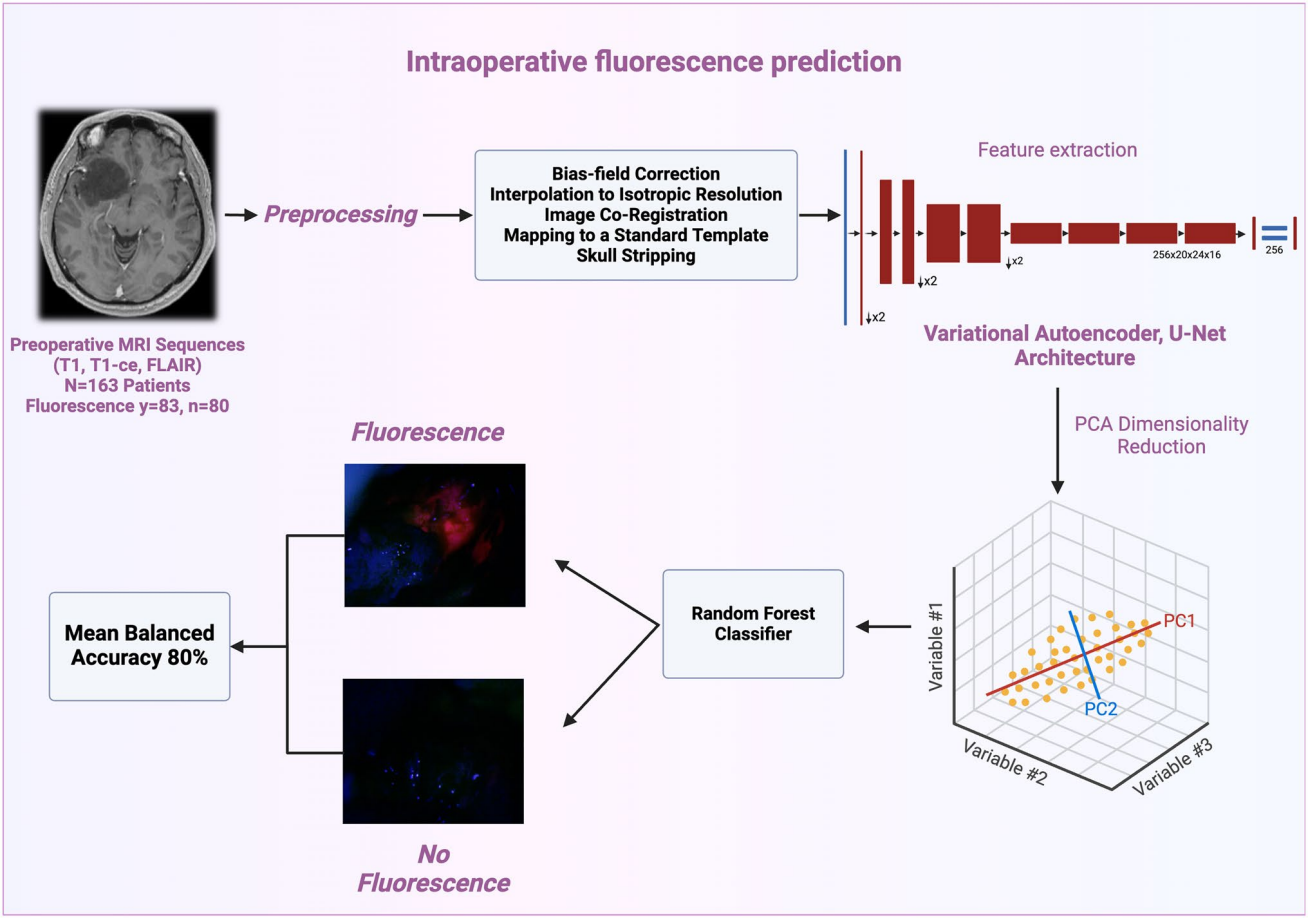
Workflow for predicting intraoperative fluorescence of gliomas with lower-grade radiographic characteristics. The model uses MRI data as input. After preprocessing and feature extraction with a deep learning model, the classification is performed via Random Forest, achieving a mean balanced accuracy of 80%

We implemented two feature selection methods—PCA and PLS-DA—to enhance classification accuracy by reducing the high dimensionality of the bottleneck features. PCA proved effective, yielding a balanced accuracy of 80% with a mean sensitivity of 84% and specificity of 76% (Fig. 3). In comparison, PLS-DA achieved a balanced accuracy of 77%, with a mean sensitivity of 75% and specificity of 76%.

To highlight the necessity of dimensionality reduction, we conducted a classification task using all 256 bottleneck features without PCA or PLS-DA. This resulted in a lower accuracy of 68%, with a sensitivity of 67.5% and specificity of 68.7%. These findings underscore the importance of dimensionality reduction techniques like PCA and PLS-DA, which enhance classification accuracy by reducing noise, concentrating on the most discriminative features, and minimizing the risk of overfitting.

### PPIX accumulation in LGG

The underlying causes behind the accumulation of PPIX in LGG tissues are poorly understood. However, numerous factors have been suggested as potential influencers. These include the tumor's proliferation and metabolic characteristics [30–32]. Additionally, conditions within the tumor microenvironment, such as hypoxia, pH levels, temperature [33, 34], and the permeability of the blood–brain barrier [18], are believed to play significant roles. Moreover, the density of tumor cells, which has been linked to visible fluorescence, demonstrates a variation between high-grade and lower-grade gliomas [35, 36]. This variation in cell density further complicates our understanding of the factors influencing PPIX accumulation in glioma tissues.

Several factors indicate a higher rate of visible fluorescence during the surgical resection of lower-grade gliomas. These include any enhancement observed with gadolinium in MRI scans, the proliferation rate indicated by the Ki-67/MIB-1 index, a high uptake value from ^18^F-fluoroethyl-tyrosine (FET) positron emission tomography (PET) scans, and the indirect measurement of tumor cell density through apparent diffusion coefficient imaging [17].

### Significance of predicting 5-ALA-induced fluorescence

Accurately predicting 5-ALA-induced fluorescence in LGGs is paramount for deciding when to use this surgical adjunct, facilitating personalized treatment planning, reducing the costs of using 5-ALA, and thus enhancing surgical outcomes [37, 38]. As described above, this approach significantly impacts the management of LGGs by enabling better identification of anaplastic foci, thereby allowing for precise resection and mitigating the risk related to undergrading. Furthermore, the benefit of having real-time information from a surgical adjunct through the routine surgical microscope view is paramount.

Areas prone to or of malignant transformation may not be apparent under conventional imaging techniques (i.e., microscopy, intraoperative ultrasound, or MRI). For instance, Widhalm et al. found that visible 5-ALA-induced fluorescence and quantitative PpIX analysis can detect focal intratumoral areas of malignant transformation, supporting maximal safe resection and increased precision of tissue sampling during surgery for suspected LGGs [39]. Moreover, Lau et al. showed a strong correlation between 5-ALA-induced fluorescence intensity and tumor cellularity [40]. Hosmann et al. suggest that intraoperative 5-ALA-induced fluorescence is an intraoperative marker of unfavorable prognosis during LGG surgery, emphasizing its role in personalized medicine [41]. Jaber et al. demonstrated that patients with Grade 2 gliomas and fluorescent tumors have an unfavorable prognosis than those without fluorescence [6].

Although fluorescence rates within LGGs are typically low [17, 21], increasing the administered dose of 5-ALA from 20 to 40 mg/kg can help overcome the blood–brain barrier (BBB) permeability in LGG [18]. Hypothetically, the higher dose increases the crossing of 5-ALA through BBB into tumor tissue, resulting in a doubled incidence of visible fluorescence [17, 21].

### Clinical implications

The implementation of predictive models marks a shift towards data-driven, personalized neurosurgery. Traditionally, decisions regarding 5-ALA administration in LGGs have been based primarily on general guidelines and the surgeon’s experience. Neurosurgeons use a specific dose and timing based on the framework from HGG treatment. With predictive modeling, these decisions can be supported by quantitative data specific to each patient's tumor characteristics, enhancing the precision of surgical interventions and supporting clinical decision-making.

Unlike traditional imaging methods such as T1-Gd contrast enhancement or DWI sequences, which may correlate with tumor cellularity or contrast uptake, our approach offers a non-invasive preoperative prediction tool that does not rely on post-surgical histological or molecular data. This enables more effective identification of fluorescence potential in lower-grade gliomas, improving decision-making for 5-ALA administration before surgery.

Furthermore, this technology could foster a more collaborative decision-making process involving multidisciplinary teams in evaluating predictive model outputs. Providing a tangible, data-based rationale for 5-ALA administration encourages consensus-building among neurosurgeons, ensuring a unified approach to patient care and supporting remuneration where it is missing.

### Limitations

The model's predictive accuracy is good but can be improved further. Although substantial for these rare tumors, the dataset's size could benefit from expansion, potentially improving the model's robustness. Additionally, the inherent heterogeneity resulting from utilizing MRI scans from 25 scanners presents a challenge, as it introduces variability that may affect the model's performance (Suppl. Table 1). However, this variability can also be viewed as a strength, as it reflects real-world diversity and may improve the model's generalizability. Furthermore, the surgical reports lacked sufficient detail to distinguish between patchy and homogeneous fluorescence. Consequently, the model was trained to detect any tumor exhibiting fluorescence, making it broadly applicable to cases that would benefit from 5-ALA injection.

While we recognize the value of model explainability techniques, such as Gradient-weighted Class Activation Mapping (Grad-CAM), exploring these methods was not an objective of this study. However, we acknowledge their importance and will consider them in future research to validate the clinical relevance of our predictions.

## Conclusions

The study used MRI and AI to predict intraoperative fluorescence in lower-grade gliomas, achieving a mean balanced accuracy of 80%. This demonstrates the potential of using deep learning for fluorescence prediction in these tumors. While the model can still be improved, it can evaluate when to administer 5-ALA to tumors lacking typical imaging features of high-grade gliomas.

## Supplementary Information

Below is the link to the electronic supplementary material.

Supplementary file1 (XLSX 12 kb)

## Data Availability

No datasets were generated or analysed during the current study.
